# Being in the zone: physiological markers of togetherness in joint improvisation

**DOI:** 10.3389/fnhum.2015.00187

**Published:** 2015-05-05

**Authors:** Lior Noy, Nava Levit-Binun, Yulia Golland

**Affiliations:** ^1^Department of Molecular Cell Biology, Weizmann Institute of ScienceRehovot, Israel; ^2^The Theatre Lab, Weizmann Institute of ScienceRehovot, Israel; ^3^Sagol Center for Brain and Mind, Baruch Ivcher School of Psychology, Interdisciplinary CenterHerzliya, Israel

**Keywords:** togetherness, joint improvisation, interpersonal synchrony, dyadic physiology, arousal, group flow, social coordination

## Abstract

Performers improvising together describe special moments of ‘being in the zone’ – periods of high performance, synchrony, and enhanced sense of *togetherness*. Existing evidence suggests a possible route for attaining togetherness – interpersonal synchrony, the fine-grained sensory-motor coordination that promotes social connectedness. Here, we investigated the physiological characteristics of togetherness using a practice from theater and dance, the *mirror game*. Pairs of expert improvisers jointly improvised synchronized linear motion, while their motion tracks and cardiovascular activity were continuously monitored. Players also provided dynamic ratings of togetherness while watching video recordings of their games. We identified periods of togetherness using kinematic and subjective markers and assessed their physiological characteristics. The kinematic and the subjective measures of togetherness showed some agreement, with more extensive game periods being marked by the subjective than the kinematic one. Game rounds with high rates of togetherness were characterized by increased players’ cardiovascular activity, increased correlation of players’ heart rates (HRs), and increased motion intensity. By comparing motion segments with similar motion intensity, we showed that moments of togetherness in the mirror game were marked by increased players’ HRs, regardless of motion intensity. This pattern was robust for the subjectively defined periods of togetherness, while showing a marginal effect for the kinematically defined togetherness. Building upon similar findings in flow research we suggest that the observed increase of players’ HRs during togetherness periods in the mirror game might indicate the enhanced engagement and enjoyment reported by performers going into ‘the zone.’ The suggested approach, combining temporal measurements of kinematic, physiological and subjective responses, demonstrates how the dynamics of spontaneously emerging dyadic states can be studied empirically.

## Introduction

Experienced musicians, actors, and dancers describe peak moments of high performance and synchrony during joint improvisation (Berliner, 1994). These moments are referred to as ‘being in the zone’ – a state of “unselfconscious awareness, in which every individual action seems to be the right one” (Seham, 2001). Subjective reports suggest a strong sense of togetherness in such moments, marked by dissolution of the self-other boundaries (Nachmanovitch, 1990; Schechner, 1994). These special moments, arising from a continuous coordinated interaction between people, are defined here as moments of togetherness. They relate to a common human phenomenon, appearing in a variety of social contexts, including collective rituals (Durkheim, 1965; Freeman, 1998; Bulbulia et al., 2013), empathic communication (e.g., Batson et al., 1991) and mother-infant relationship (e.g., Trevarthen, 1979; Feldman, 2006).

Joint improvisation can be viewed as a special case of *joint action*, the dynamic coordination between individuals aimed at bringing a change in the environment (Sebanz et al., 2006). A completion of any joint action, whether dancing or moving a heavy object together, is crucially dependent upon the fine-grained temporal coordination between individuals. Recent research has elucidated a potent mechanism subserving interpersonal coordination – the spontaneous emergence of sensory-motor synchronization between interacting individuals (see Knoblich et al., 2011; Repp and Su, 2013 for review). Examples of emerging interpersonal synchrony include synchronization of body postures during communication (Shockley et al., 2003), and the tendency of people walking together to synchronize limb swinging (Mottet et al., 2001). Interpersonal synchrony has been suggested to act as a ‘social glue,’ binding individuals together into a larger whole (e.g., Wilson et al., 2008), eliciting sense of connectedness, compassion, and rapport (Hove and Risen, 2009; Valdesolo et al., 2010; Valdesolo and DeSteno, 2011). Interpersonal synchrony, characterized by fine-grained coordination and a sense of connectedness, can be considered as a mechanism subserving togetherness in joint improvisation.

The majority of studies investigating interpersonal synchrony have mainly focused on individuals coordinating repetitive, rhythmic behaviors, such as tapping. Research that examined complex forms of coordination mostly studied music performance, in which individuals synchronize with external rhythm (for review see Repp and Su, 2013). In joint improvisation, as in real-life, people exhibit more complex patterns of coordination, which can also arise spontaneously without a pre-defined script. Building on the growing body of research in interpersonal synchrony, we recently developed an experimental paradigm to study togetherness in an open-ended joint improvisation task. We employed the mirror game, a practice from theater and dance in which two actors mirror each other, creating synchronized dance-like motions together (Noy et al., 2011; Hart et al., 2014). In the mirror game expert improvisers can enter a unique dyadic state where they create especially synchronized and complex motions, without a leader or follower. We suggested that these motions can serve as a kinematic marker of togetherness.

In the current work, we employ the mirror game paradigm to investigate whether periods of togetherness in joint improvisation have a distinctive physiological underpinning. The physiology of togetherness states is under-studied, in particular in open-ended tasks. Here, we measured the cardiovascular activity of players in the mirror game. Cardiovascular activity is one of the common indices of the autonomic nervous system (ANS) – a general-purpose physiological system that adapts its activity to the altered needs of the organism (Critchley et al., 2005). In particular, cardiovascular arousal has been associated with states eliciting arousing positive emotions, mental effort and active coping (Bradley and Lang, 2000; Critchley et al., 2000, 2005; Codispoti et al., 2001; Wright and Kirby, 2001; Richter et al., 2008; Vaez-Mousavi et al., 2009; Kreibig, 2010).

Peak moments of joint performance are highly engaging and rewarding, and evoke intense positive emotions (Berliner, 1994, p. 389). Research suggests that interpersonal synchrony also elicits positive emotional states and involves the engagement of neural reward circuitry (Kokal et al., 2011). We therefore hypothesize that periods of togetherness during joint improvisation in the mirror game will be characterized by increases of players’ heart rates (HRs).

In addition, it has been suggested that enhanced social coordination involves synchronization of physiological activity among interacting individuals (Butler, 2011; Hasson et al., 2012; Konvalinka and Roepstorff, 2012). For example, oscillatory cac and respiratory patterns was observed between singers and a conductor in a choir (Müller and Lindenberger, 2011), and between performer and related observers in a real-life fire ritual (Konvalinka et al., 2011). Similar findings were found in the neural domain (Babiloni and Astolfi, 2012; Konvalinka and Roepstorff, 2012). Such synchronization of activity has been suggested to arise as a result of mutual and adaptive coordination between individuals during interaction, leading to shared behavioral and physiological states (Konvalinka and Roepstorff, 2012). Building upon this framework, we suggest that during periods of togetherness in the mirror game both the behavioral and the physiological systems of the two players become tightly coupled. We therefore hypothesize that these periods will be associated with increased correlation of players’ HRs.

**FIGURE 1:**
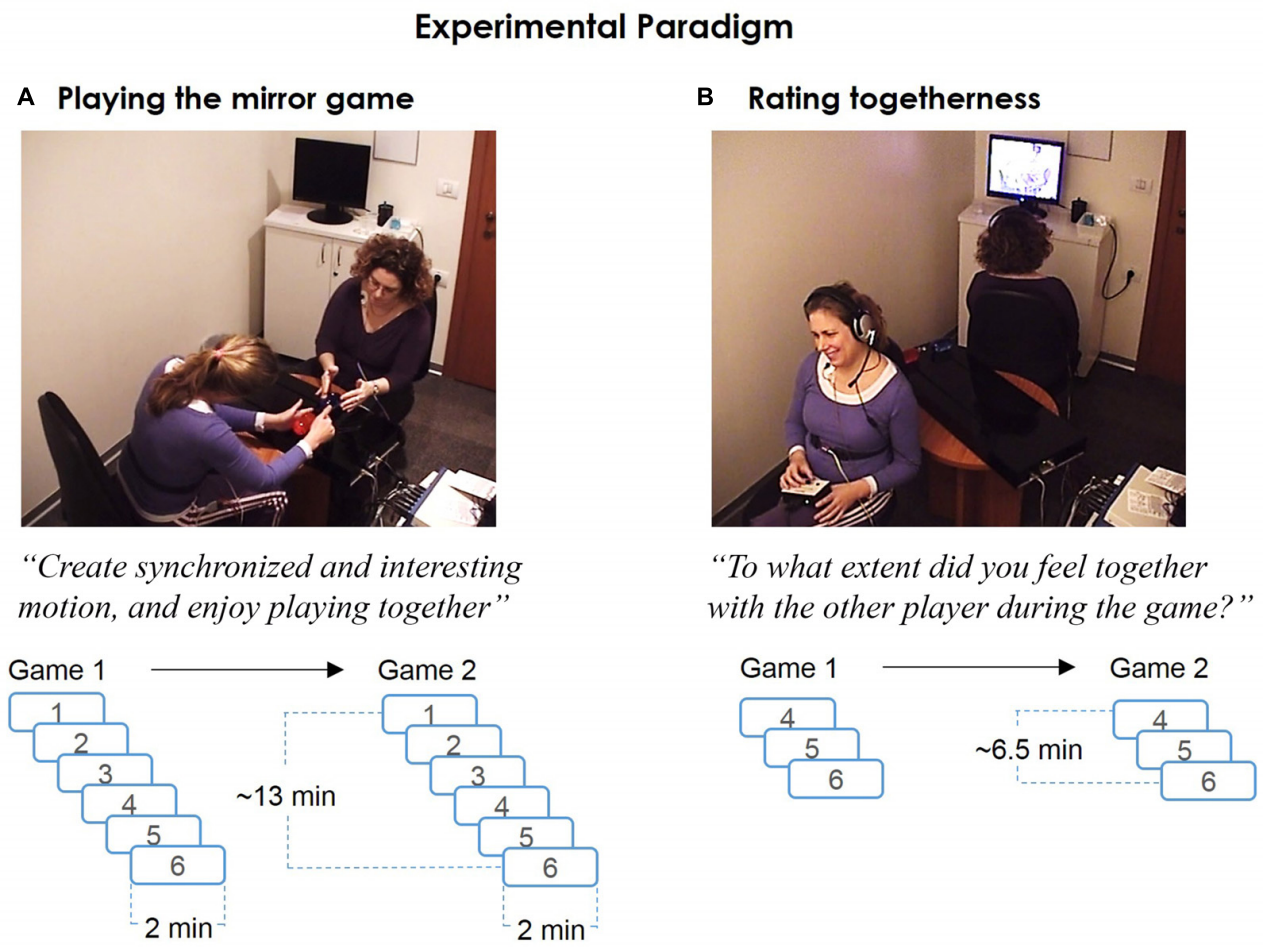
**The experimental paradigm. (A)** Playing the mirror game. In the mirror game players moved handles along parallel tracks, improvising “synchronized and interesting motion” together. Each pair played two games, consisting of six 2 min rounds. Dyadic physiological measurements (ECG, 1 KHz) of the two players were temporally synchronized with the motion tracking (50 Hz). Videos of the whole game were recorded by two pre-installed cameras. **(B)** Rating togetherness. Immediately after the game, participants were presented with the video recordings of the last three rounds from each game. They provided continuous subjective ratings (SR) of togetherness during the game, using a rating-dial device (1 KHz).

**FIGURE 2:**
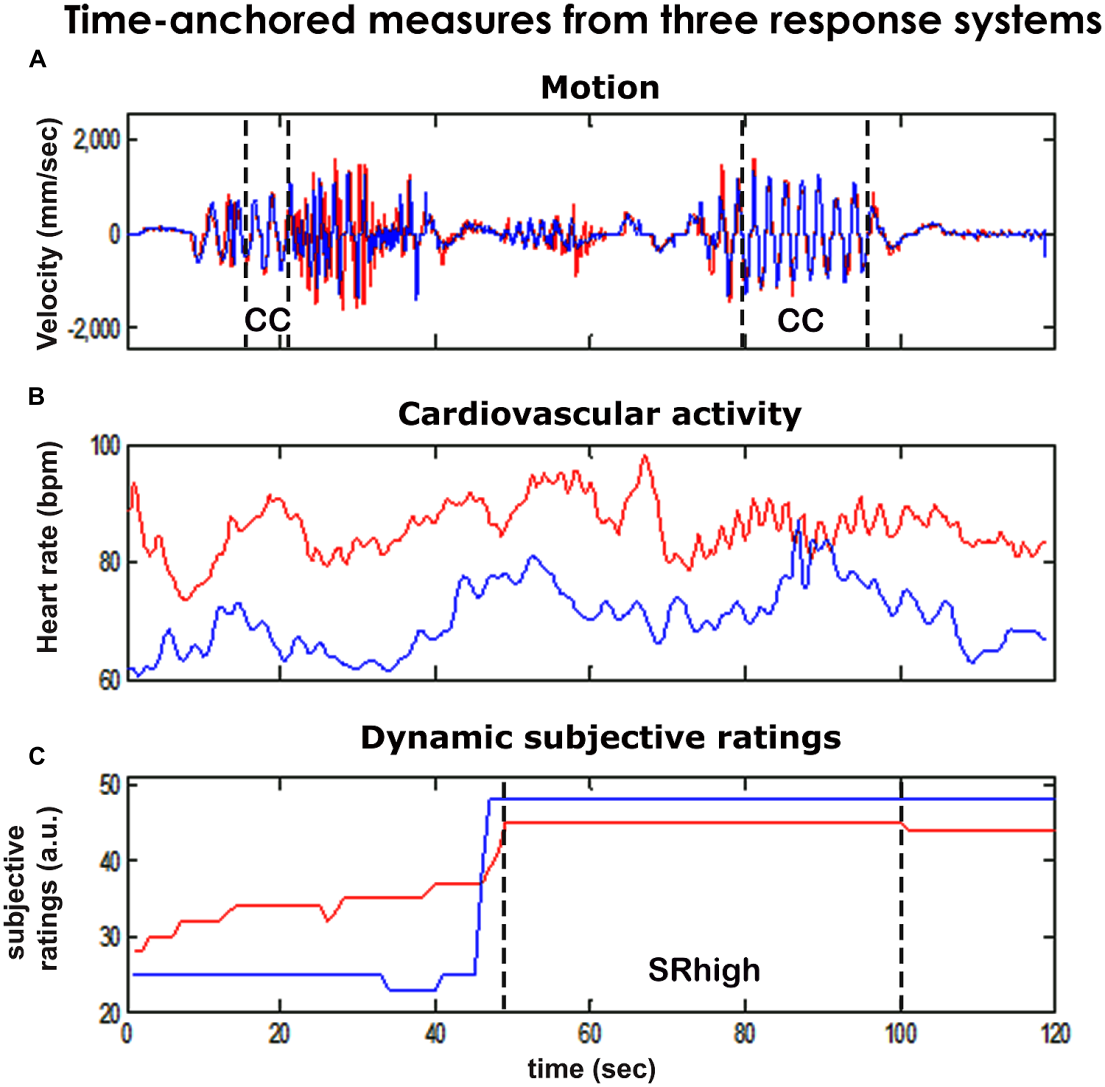
**Time anchored measures from three response systems.** We collected continuous, temporally anchored measures from three response systems of the two players in the mirror game (red and blue lines). We defined periods of togetherness by using kinematic and subjective markers and studied the physiological characteristics associated with these periods. **(A)** Motion. Using analysis of motion tracks (50 Hz), we identified periods of kinematic togetherness (Noy et al., 2011), that is periods of especially synchronized, smooth motion co-confident motion (CC). Kinematic data also allowed for the assessment of motion intensity characteristics (maxVel, Freq). **(B)** Physiological activity. Continuous (2 Hz) measures of players’ heart rates (HRs) were derived from ECG recordings. We calculated mean players’ heart rates (HR) and correlation of players’ HRs (corrHR). **(C)** Subjective ratings. We identified periods of subjective togetherness (SRhigh), as periods that were rated as high (top 30% across games) by both players.

To test these hypotheses we developed a setup incorporating three measures of joint motion improvisation: kinematic, physiological and subjective assessment (**Figure 1**). Motion traces were measured to detect periods of kinematic synchrony and other kinematic variables (**Figure 2A**). Cardiovascular arousal and inter-player HR correlation were quantified from players’ cardiovascular activity (**Figure 2B**). In addition, post-game subjective reports provided a subjective marker of togetherness (**Figure 2C**). We hypothesized that periods marked by enhanced subjective togetherness or kinematic synchrony will be accompanied with increased cardiovascular activity and inter-player HR correlation.

## Materials and Methods

### The Mirror Game Setup

A custom-made device was developed to allow motion measurement in the 1D mirror game (see Noy et al., 2011). Briefly, a pair of expert improvisers moved handles along parallel tracks (see Supplementary Figure S1). They were instructed to produce mirror-like motion that is ‘synchronized and interesting.’ The motion of the two handles was sampled at 50 Hz. There were two types of rounds: in leader–follower (LF) rounds, a designated follower imitated a designated leader’s motion. In joint-improvisation (JI) rounds, the motion was created by the pair of players without a designated leader or follower. A set of colored lights indicated the type of round. A bell sound marked the start and the end of each round. Rounds were separated by 10 s rest periods. During the game players sat facing each other, holding the handles comfortably with both hands. At times, players discovered other ways to move the handles such as holding the handle with a single hand, pushing it gently with the fingers or hitting it back-and-forth to produce rapid motions. The setup was integrated with a dyadic physiological measurement setup (Mindware Technology, Gahanna, OH, USA), with both systems being temporally synchronized via a dedicated TTL trigger (Supplementary Figure S1).

### Pilot Study

A pilot study was conducted to test the feasibility of the setup. The goals of the pilot study were to test: (1) whether players can produce profound joint improvisation behavior in the lab, while their HR is being monitored; (2) whether physiological activity could be reliably recorded during motion improvisation.

Three pairs of expert improvisers played two games each. The first game followed a previously published (Noy et al., 2011) game structure [nine 1 min rounds, alternating between Leader-Follower (LF) and Joint-Improvisation (JI) rounds]. The second game consisted of four 2.5 min JI rounds. Out of three pairs of experts who participated in the pilot study, two showed a fair rate of Co-confident motion (CC motion, Noy et al., 2011 and see below) (0.09 and 0.23, see **Table 1**), and their physiological recordings could be analyzed and interpreted. Thus, the pilot study supported the feasibility of the proposed experimental setup.

**Table 1:** Game characteristics for each pair of players.

Pair	Gender	# rounds	# SR rounds	CC rate	SRhigh rate	corrHR	HR (bpm)	maxVel (mm/s)	Freq (Hz)
*1*	*F* *M*	6	3	0.05 ± 0.05	0.3 ± 0.3	0.36 ± 0.18	83 ± 2.7 68 ± 3.3	414 ± 222 360 ± 198	0.34 ± 0.18 0.32 ± 0.17
*2*	*F* *F*	7	4	0.02 ± 0.02	0.17 ± 0.1	0.65 ± 0.17	75 ± 7.2 93 ± 11	203 ± 150 153 ± 153	0.39 ± 0.3 0.32 ± 0.33
*3*	*F* *F*	12	6	0.01 ± 0.02	0.22 ± 0.16	0.52 ± 0.25	82 ± 3.1 83 ± 4.3	169 ± 114 160 ± 99	0.18 ± 0.09 0.17 ± 0.09
*4*	*F* *F*	10	5	0.08 ± 0.04	0.11 ± 0.09	0.38 ± 0.24	87 ± 3.8 72 ± 3.2	266 ± 74 279 ± 64	0.19 0.04 0.21 ± 0.05
*5*	*F* *M*	4	4	0.09 ± 0.09	0.27 ± 0.31	0.5 ± 0.36	85 ± 3.2 79 ± 1.6	278 223 339 301	0.39 0.28 0.32 0.22
*6*	*M* *F*	4	4	0.26 ± 0.15	0.34 ± ± 0.25	0.3 0.11	62 ± 2.8 85 ± 1.0	411 ± 182 432 ± 207	0.42 ± 0.18 0.39 ± 0.15
*7*	*M* *M*	10	4	0.07 ± 0.05	0.17 ± 0.20	0.4 ± 0.24	73 ± 1.3 77 ± 3.2	353 ± 248 359 ± 256	0.4 ± 0.38 0.41 ± 0.33
*8*	*F* *M*	12	6	0.01 ± 0.01	0.11 ± 0.05	0.32 ± 0.18	93 ± 1.4 85 ± 6.2	101 ± 95 99 ± 85	0.16 ± 0.13 0.18 ± 0.10
*9*	*M* *F*	12	6	0.11 ± 0.08	0.18 ± 0.18	0.2 ± 0.19	77 ± 2.3 78 ± 2.9	236 ± 146 235 ± 134	0.34 ± 0.20 0.38 ± 0.20

The table presents the number of valid rounds (#rounds), that is, rounds in which intact physiological and kinematic recordings were available for analysis; gender of each player in the dyad (*F*-female, *M*-male); the number of valid rounds in which players’ subjective ratings were obtained (#SR rounds), as well as mean scores (across all game rounds for each pair) and standard deviations of the following measures: CC rate, rate of CC motion (kinematic togetherness) in each round; SRhigh rate, rate of SRhigh (subjective togetherness) in each round; corrHR, within dyad heart rate correlation; HR, median heart rate; maxVel, weighted median of maximal velocity; Freq, weighted median of frequency. HR, maxVel, and Freq are individual measures; CCrate, SRhigh rate, and corrHR are dyadic measures (one line per pair).

### Main Study Participants

Nine pairs of experts, having at least 5 years’ experience in joint motion improvisation participated in the experiment. Participants were recruited through advertisements in social networks and were paid for participation. The study was approved by the Interdisciplinary Center research ethics committee (IDC IRB), and written informed consent was obtained after the procedures had been fully explained.

### Experimental Procedure

#### Introduction and Hook Up

Players were introduced to the experimental setup, were seated facing each other and were given an explanation about the experiment procedure. Players were instructed that the goal of the game is to “create synchronized and interesting motions, and enjoy playing together.” The experimenter emphasized that the game is not a competition, and that the aim is to enjoy creating motion together. Following the initial instructions players were hooked to the physiological sensors.

#### Stage1 – Mirror Game

Before the actual game, a practice game consisting of three 15 s rounds was used to acquaint players with the procedure. After the practice game, the experimenter answered any additional questions about the game procedure and left the room until the end of this stage. Each pair of players played two games, each consisting of six 2 min rounds. A game started with two LF rounds (with alternating leaders) and continued with four JI rounds (**Figure 1A**).

#### Stage2 – Subjective Ratings

After playing the mirror game, the two players separately watched video recording of the game and provided continuous subjective ratings (SR) of the extent they felt together with the other player during the game (**Figure 1B**). They were seated back-to-back, in front of two computer screens, with earphones. Video recordings from the three last rounds of each game (overall six rounds) were presented. Players were asked to rate the degree of togetherness they felt with the other player during the game, using a rating dial. The rating dial spans a 180° arc, with 180° signifying “extremely high levels of togetherness,” 0° signifying “no togetherness” and 90° signifying neutral level of togetherness. Players were asked to initiate the ratings of each round at 90° and provide their ratings relative to this level. At the end of the experiment players were debriefed and any additional questions were answered.

### Final Dataset

Following our previous experience (Noy et al., 2011) we aimed to collect data from nine pairs of expert improvisers. Of the nine pairs, two pairs were removed due to noisy electrocardiogram (ECG) signal from one of the players, prohibiting a reliable extraction of the cardiovascular time-series. In the remaining seven pairs, 15 of the 84 rounds in the dataset (17.8%) were removed, either due to missing motion data (five rounds, 5.9% of all rounds) or gross artifacts in the physiological signals (10 rounds, 11.9% of all rounds). To accommodate for the loss of power due to missing data, we included two pairs from the pilot study in the final data-set. To match the duration of pilot rounds to that of the new pairs, we included only the second game rounds of the pilot study participants, and only the first 2 min of each round. The pilot study participants where re-invited to the lab and provided SR for the four JI rounds from the second game. The final dataset contained nine pairs and 77 game rounds. The numbers of included rounds as well as rounds’ descriptive statistics are reported in **Table 1**. The final dataset of nine pairs of expert improvisers contained seven men and eleven women (age 36.2 ± 6.4), fifteen of which were right-handed. Five pairs had a female player and a male player, three pairs had two female players and one game had two male players (see also Supplementary Figure S4).

### Physiological Recording and Preprocessing

Cardiovascular activity of the two players was recorded with an integrated system and software package (Mindware Technology, Gahanna, OH, USA) at a sample rate of 1 kHz. Recording was initiated by the TTL signal sent from the mirror game device to ensure synchronization of the physiological and kinematic signals (Supplementary Figure S1). Cardiovascular responses were recorded with the ECG amplifier module. Alcohol pads were used to clean the skin sites and cotton pads to dry them. Electrocardiography was recorded using three disposable pre-gelled Ag/AgCl spot electrodes positioned in a three-lead unipolar modified chest configuration: the two active electrodes were placed on the right collar bone and the lowest rib on the left side, and the ground electrode was placed on the left collar bone. The heart period [inter-beat interval (IBI)] was assessed using Mindware HRV 2.16 bio-signal processing module by (a) identifying the R–R intervals (b) detecting physiologically improbable R–R intervals based on the overall R–R distribution using a validated algorithm (Berntson et al., 1990); and (c) manually inspecting the data to ensure that R-waves were correctly identified. Data segments, in which Rs could not be correctly identified (the QRST complex could not be detected) due to artifacts, were replaced with missing values. Rounds containing more than 10% missing values were excluded from the analysis (overall 10.9% of rounds removed). IBI series were transformed to continuous 2 Hz HR time-series using an interpolation algorithm.

### Video Recording

Audio and video recordings of the first stage of the experiment (playing the mirror game) were made using two pre-installed cameras. Each camera captured the front plane of each player and the mirror game device. These recordings were shown to the players during the second stage of the experiment. A video frame example can be seen in **Figure 1A**.

### Subjective Ratings Recording and Preprocessing

The continuous subjective ratings (SR) were recorded using a rating dial (Mindware Technology, Gahanna, OH, USA). The output of the rating dial yielded near continuous data (1 kHz) that was resampled to 1 Hz using custom made software. To fix video recording timing measurement errors (range [0–10] s), SR of each round were interpolated to 120 s length.

### Data Analysis

#### Detecting CC Periods

We used the previously developed notion of CC motion, defined as periods of high synchrony with little jitter (Noy et al., 2011). We developed a new CC detection algorithm. Segments were considered as part of a CC period if they matched two conditions: (1) they contained exactly one acceleration zero crossing (that is, a single velocity peak, with no jitter), (2) the segments of the two players were fairly similar. The similarity condition was included to remove segment pairs that were both smooth, but were too different to be considered synchronized. Segment pairs were removed if (1) the distance of their stopping-point events were larger than 0.15 s, if (2) the distance of their peaks events were larger than 0.3 s, or if (3) the normalized velocity error (dV from Noy et al., 2011) between the two segments was larger than 0.95. These thresholds were set to maximize correct detection rate on a set of segments manually labeled. The final correct detection rate was ∼90%, compared to ∼80% of the previous algorithm applied to the same sample of the current dataset. A main source of errors was missed CC periods in low velocities (<500 mm/s), due to noise in the velocity signals.

#### Kinematic Measures

On average ∼67% of each round was covered with motion segments. Motion segments were periods of motion between two zero velocity events, longer than 0.2 s and shorter than 8 s. 95% of segments were below 2.8 s, with mean = 0.83 s, SD = 0.93. Two measures of motion intensity were computed for each motion segment: frequency (Freq) and maximal velocity (maxVel). Frequency was defined as half the inverse of segment duration, considering each velocity segment as a half-period (Freq = 0.5 ^∗^ 1/duration). maxVel was smoothed by taking the median of the top 10% values in each segment [maxVel = median (Top10%(velocity-segment))]. In addition, each motion segment was indexed as CC or non-CC by the automatic CC detector algorithm.

For the round level analysis, we calculated for each game round the median Freq and maxVel (weighted by segments’ duration) as well as the duration of the round covered with CC segments, divided by the total round duration (CCrate). No differences were observed between LF and JI rounds in these measures (Supplementary Figure S2).

#### Physiological Measures

For the round level analysis, arousal was calculated as a median value of individual HR time-series (HR) in each round. Physiological coupling (corrHR) was calculated as Pearson correlation between the two players’ HR time-series in each round. For the segment level analysis, which pools across players, games and rounds, the HR scores of each player were z-normalized across all data points of that player, to remove individual differences in basal HR. Segment’s arousal was indexed as average zHR in that segment.

#### SR Measures

For each rated game round we computed the proportion of time in which both players provided high rates of togetherness (SRhigh periods). High rates of togetherness were set individually to account for individual differences in the range of the dial responses (see Supplementary Figure S3). A threshold was set for each player to the top 30% across all data points (changing the threshold to 25% or 20% provided qualitatively similar results).

Motion segments were marked as SRhigh if more than 75% of their sample points were within a SRhigh period, and as non-SRhigh otherwise. Using the SR indexes, we compared players’ physiological activity, that was recorded during the game, for segments which were rated as SRhigh and non-SRhigh.

## Results

### Expert Improvisers can enter Periods of Togetherness while their Heart Rate is being Monitored

The present dataset includes 77 game rounds played by nine pairs of players. For each game round we calculated a series of kinematic, physiological and subjective measures. Motion intensity was assessed by the maximal velocity (maxVel) and frequency (Freq) of the motion traces, computed per segment and aggregated across rounds’ segments (see Materials and Methods). Physiological characteristics of the game rounds were indexed by players’ mean HR and correlation of players’ heart rates (corrHR).

**FIGURE 3:**
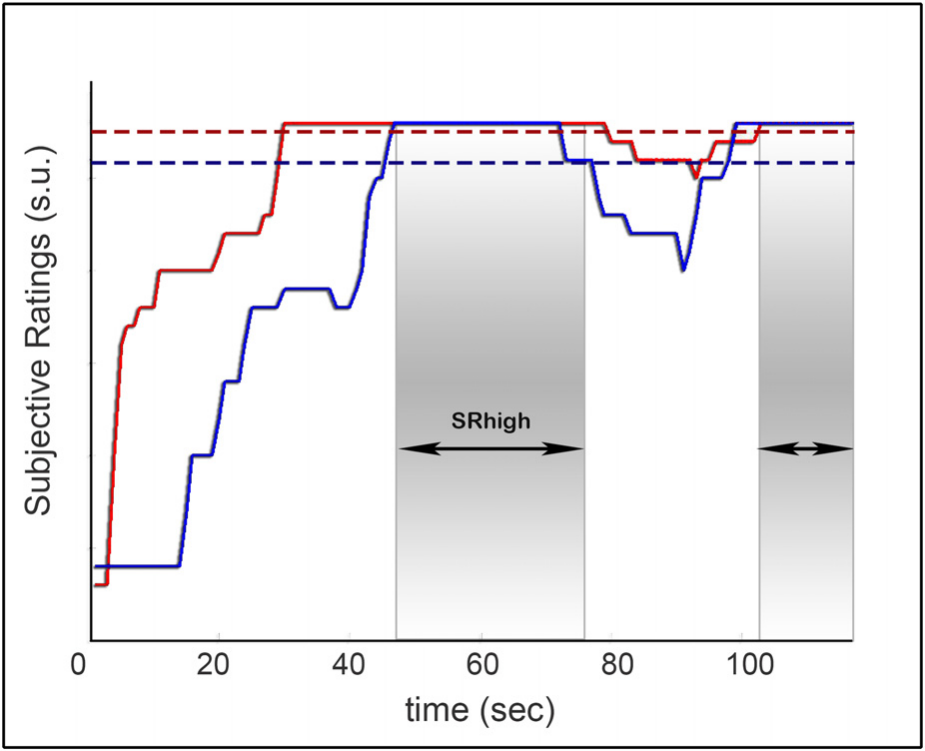
**Identifying subjective togetherness. SR for one game round provided by two players (red and blue lines).** The thresholds for high togetherness (top 30% across all game rounds) are marked by red and blue dotted lines. Game periods, in which both players provided above threshold responses, were indexed as high togetherness (SRhigh, gray regions).

In addition, we assessed rates of kinematic and subjective togetherness for each game round. Periods of kinematic togetherness were defined as periods of co-confident (CC) motion – smooth and synchronized motion (see Materials and Methods). CC motion comprised on average 0.07 (SD = 0.08) of the rounds’ duration. Subjective togetherness was defined using players’ post-game SR of experienced togetherness in the game (**Figures 1B** and **2**, lower panel). The SR dataset included 42 game rounds played by nine pairs of players. The overall agreement in the SR between the two players was moderate (average Pearson correlation over all SR rounds = 0.51, SD = 0.39). Often, one player provided more conservative scores than the other, hence we assumed that the dyadic measure would serve as a better estimate of subjective togetherness (see Supplementary Figure S3 for qualitative examples). Accordingly, game segments in which both players’ SR scores were higher than the top 30% were marked as periods of subjective togetherness (SRhigh, see Materials and Methods, **Figure 3**). On average, a ratio of 0.19 (SD = 0.18) of round duration was marked as SRhigh periods.

**Table 1** presents descriptive statistics of the kinematic, physiological and subjective measures computed for each pair of players in the current dataset.

There were some differences between mixed-gender and same-gender games, with female–female pairs showing higher corrHR and lower CCrate than female–male pairs (see Supplementary Figure S4). These results are not discussed further due to the small number of participating pairs in this study.

### Game Rounds with High Rate of Kinematic and Subjective Togetherness are Characterized with Increased Players’ HRs, Higher Inter-Player HR Correlation and Higher Motion Intensity

To assess the relationship between rate of togetherness, physiological characteristics and motion intensity, we computed rank correlations between these measures for each pair of players. **Table 2** presents a group-level analysis, in which we calculated the average correlation coefficients across the nine pairs and assessed its statistical likelihood using non-parametric bootstrapping, by randomly shuﬄing the order of rounds within pairs (Supplementary Figures S5 and S6 present the empirical control distributions for these tests).

**Table 2:** Rank correlations between rate of kinematic (CC) and subjective (SRhigh) togetherness, physiological characteristics (HR, corrHR), and motion intensity (maxVel, Freq).

	HR	corrHR	maxVel	Freq
SRhigh rate	0.44 ± 0.14, *p* < 0.003	0.38 ± 0.17, *p* < 0.01	0.1 ± 0.21, *p*= 0.31	0.32 ± 0.21, *p* < 0.03
CC rate	0.54 ± 0.09, *p* < 0.001	0.39 ± 0.16, *p* < 0.005	0.57 ± 0.08, *p* < 0.001	0.62 ± 0.07, *p* < 0.001
HR		0.45 ± 0.15, *p* < 0.001	0.38 ± 0.13, *p* < 0.01	0.43 ± 0.12, *p* < 0.001
HRcorr			0.39 ± 0.14, *p* < 0.01	0.53 ± 0.1, *p* < 0.001
maxVel				0.78 ± 0.04, *p* < 0.001

Rank correlations between the measures were calculated for each pair of players and averaged across pairs. Table presents the average correlation, standard error, and empirical *p*-values for each comparison.

As can be seen in **Table 2**, CC rate was significantly correlated with both physiological measures (CC-HR: *r* = 0.54 ± 0.09 SE, *p* < 0.001; CC-corrHR: *r* = 0.39 ± 0.16 SE, *p* < 0.005) as well as with the level of motion intensity (CC-maxVel: *r* = 0.57 ± 0.08 SE, *p* < 0.001; CC-Freq: *r* = 0.62 ± 0.07 SE, *p* < 0.001). In other words, during game rounds characterized with high kinematic togetherness, players exhibited higher HRs and their HRs were more correlated with each other. In addition, in these game rounds players reached higher motion velocities and frequencies.

A similar pattern was observed for the rate of subjective togetherness (SRhigh-HR: *r* = 0.44 ± 0.14 SE, *p* < 0.003; SRhigh-corrHR: *r* = 0.38 ± 0.17 SE, *p* < 0.012). In other words, in rounds that contained larger periods of high subjective togetherness, players HRs increased and were more correlated. These rounds also tended to exhibit higher motion frequencies (SRhigh-Freq: *r* = 0.32 ± 0.21 SE, *p* < 0.03), while the dependency with motion velocity was not significant (SRhigh-maxVel: *r* = 0.1 ± 0.21 SE, n.s.).

### Players’ Heart Rates Increase in Togetherness Periods, Controlling for Motion Intensity

The positive correlation of the two measures of togetherness (CC rate and SRhigh rate) with the two physiological measures (HR and corrHR) suggests that periods of togetherness in the mirror game are characterized by an increase in players’ HRs and stronger inter-player alignment of their cardiovascular activity. However, rounds with higher rates of togetherness are also characterized by more intense motion, as evident by the positive correlations of CC rate and SRhigh rate with maxVel and Freq.

**FIGURE 4:**
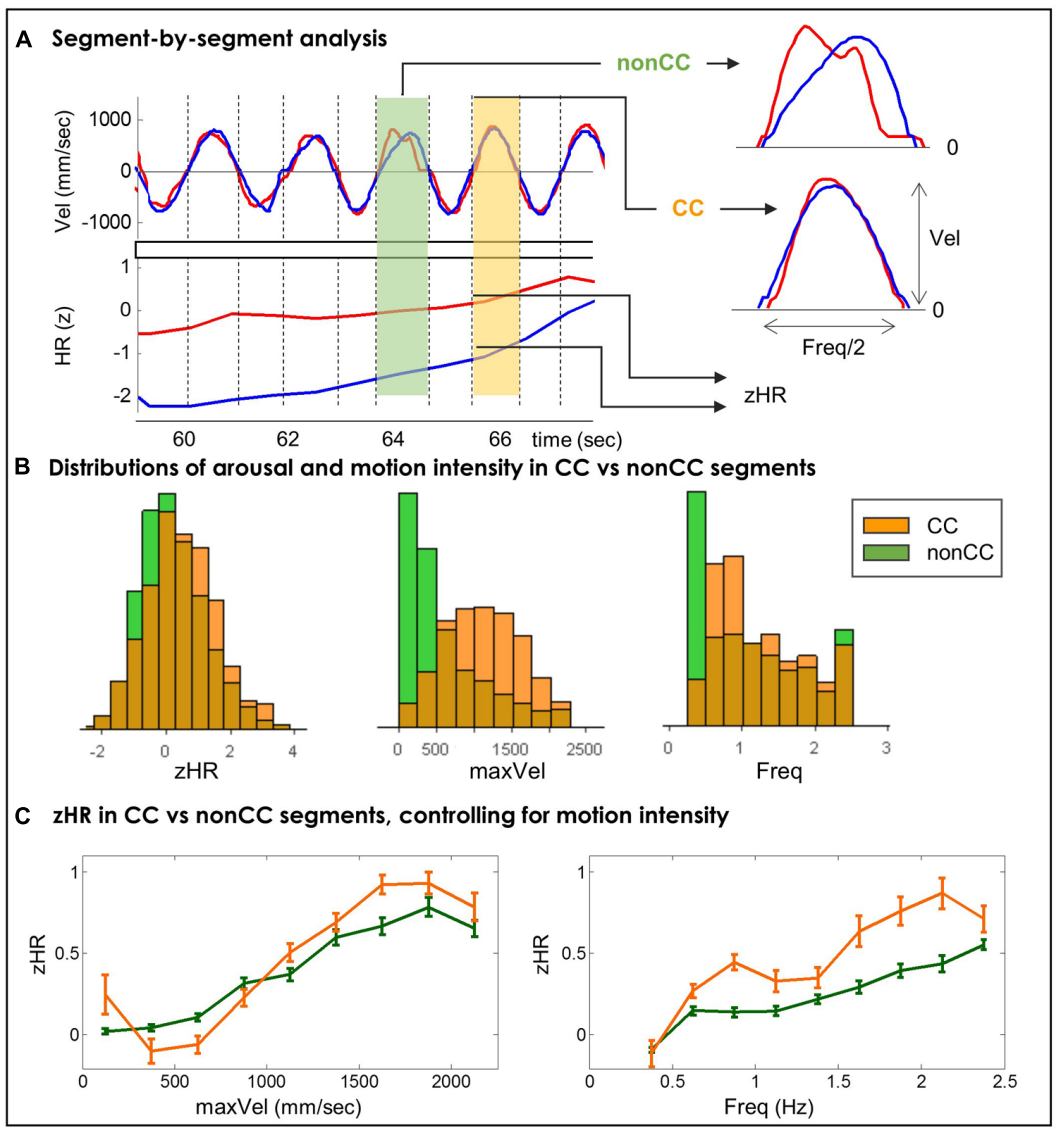
**(A)** A Segment-by-segment analysis. Motion segments are defined as periods between zero velocity events. For each segment we computed its motion intensity (Freq and MaxVel), its physiological arousal (zHR) and a binary marker of kinematic togetherness (CC or non-CC). **(B)** Distributions of players’ HRs and motion intensity in CC and non-CC segments. In CC segments compared to non-CC segments, players demonstrate higher z-normalized heart-rates, and move at higher velocities and higher frequencies. **(C)** zHR in CC vs. non-CC segments, controlling for motion intensity. Average zHR is compared for CC and non-CC segments in same bins of maxVel (left) and Freq (right). In eight out of nine bins of frequency (**Table 3**) and in one out of nine bins of velocity (**Table 4**), zHR in CC segments was significantly larger than zHR in non-CC segments.

**FIGURE 5:**
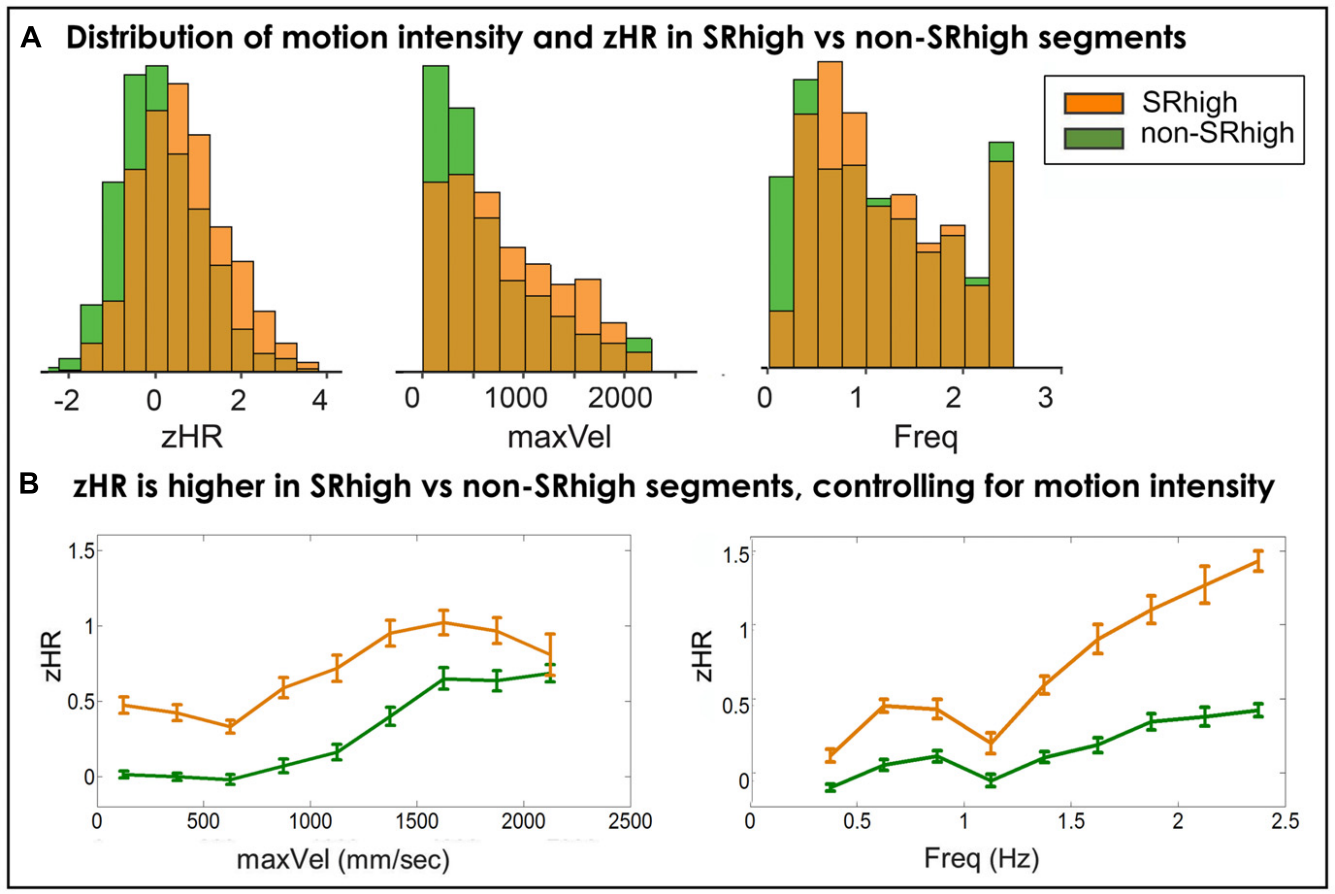
**(A)** Distributions of players’ HRs and motion intensity in SRhigh and non-SRhigh segments. In SRhigh segments compared to non-SRhigh segments, players demonstrate higher Z-normalized heart-rates, move at higher velocities and higher frequencies. **(B)** zHR in SRhigh vs. non-SRhigh segments, controlling for motion intensity. Average zHR is compared for SRhigh and non-SRhigh segments in same bins of maxVel (left) and Freq (right). zHR was higher in SRhigh segments than in non-SRhigh segment, for all frequency bins (**Table 5**) and for eight velocity bins (**Table 6**).

To control for the possible effect of motion intensity on the cardiovascular activity we assessed players’ HRs in motion segments with similar kinematic characteristics. Each motion segment was marked as CC or non-CC segment (**Figure 4A**) and as SRhigh or non-SRhigh segment. In addition, we calculated for each segment its motion intensity (maxVel and Freq) and the average of player’s normalized HR during the segment’s duration (zHR; see Materials and Methods and **Figures 4** and **5**). We analyzed players’ zHRs in nine separate bins of motion intensity.

#### Segment-by-Segment Analysis of Heart Rate and Kinematic Togetherness (CC)

We assessed the difference in zHR between CC and non-CC segments with a similar motion intensity. Pooling together across all players and rounds resulted in N_CC_ = 2,351 CC segments and N_nonCC_ = 12,298 non-CC segments (16% and 84% of total segments, respectively). **Figure 4B** presents the univariate distributions of maxVel, Freq, and zHR values for CC and non-CC segments. In CC segments compared to non-CC segments, players move at higher velocities (CC = 1115.8 mm/s, non-CC = 599.2 mm/s, non-parametric *p* < 10^-4^), and higher frequencies (CC = 1.25 Hz, non-CC = 1.11 Hz, non-parametric *p* < 10^-4^); and show higher z-normalized HRs (CC = 0.44, non-CC = 0.17, non-parametric *p* < 10^-4^).

**Figure 4C** shows average zHR for CC and non-CC segments in nine bins of maxVel and nine bins of Freq. We assessed the statistical significance of the difference in zHR between CC and non-CC segments in the same Freq and maxVel bins. We used non-parametric bootstrapping to overcome the inherent dependence between consecutive segments and dyads of players. In each motion bin we compared the actual mean-difference of the two groups [mean(zHR(CC)) – mean(zHR(non-CC)] to a distribution of mean differences of random bootstrapped samples. This distribution was created by choosing (with replacement) N_CC_ segments for a simulated “CC” group and N_nonCC_ segments for a simulated “non-CC” group, chosen regardless of their actual CC tags, and computing the mean for each group. Repeating this procedure 10,000 times resulted with a distribution of mean differences. The empirical *p*-value of the actual observed mean-difference was estimated directly from this control distribution (see Supplementary Figures S7 and S8). The resulting empirical *p*-values were subjected to multiple-comparison correction (Benjamini–Hochberg (BH), q(FDR) = 0.05).

In eight of the nine Freq bins zHR was significantly higher in CC vs. non-CC segments. Six of the nine maxVel bins showed a trend for the hypothesized effect (*p* < 0.11), however, only one maxVel bin showed significant difference, after correcting for multiple comparisons. The ranges of maxVel and Freq, the number of segments, the mean and SE of zHR and the resulting statistical test for each bin appear in **Table 3** for Freq and **Table 4** for maxVel.

**Table 3:** zHR in CC and non-CC segments, compared over similar frequencies (Freq).

Range of Freq (Hz)		0.25–0.5	0.5–0.75	0.75–1	1–1.25	1.25–1.5	1.5–1.75	1.75–2	2–2.25	2.25–2.5
CC	#seg	126	477	448	222	324	186	203	129	236
	zHR ± SE	-0.12 ± 0.08	0.27 ± 0.04	0.44 ± 0.05	0.33 ± 0.07	0.35 ± 0.06	0.64 ± 0.09	0.76 ± 0.09	0.87 ± 0.10	0.71 ± 0.08
Non-CC	#seg	3464	1410	1169	1172	1378	842	888	526	1449
	zHR ± SE	- 0.09 ± 0.02	0.15 ± 0.03	0.14 ± 0.03	0.14 ± 0.03	0.22 ± 0.03	0.29 ± 0.04	0.39 ± 0.04	0.44 ± 0.05	0.55 ± 0.03

*p*		0.615	0.009^∗^	<0.001^∗^	0.007^∗^	0.021^∗^	<0.001^∗^	<0.001^∗^	<0.001^∗^	0.029^∗^

The table presents number of segments, average zHR, and standard errors for CC and non-CC segments, in each range of motion frequencies. ^∗^Significant after correction for multiple comparisons [q(FDR) = 0.05].

**Table 4:** zHR in CC and non-CC segments, compared over similar velocities (maxVel).

Range of maxVel (mm/s)		0–250	250–500	500–750	750–1000	1000–1250	1250–1500	1500–1750	1750–2000	2000–2250
CC	#seg	81	187	353	393	399	379	306	164	89
	zHR ± SE	0.25 ± 0.12	-0.1 ± 0.07	-0.06 ± 0.05	0.23 ± 0.05	0.5 ± 0.06	0.69 ± 0.05	0.92 ± 0.06	0.93 ± 0.07	0.79 ± 0.09
Non-CC	#seg	4045	3091	1691	999	822	573	438	306	333
	zHR ± SE	0.02 ± 0.02	0.04 ± 0.02	0.11 ± 0.02	0.31 ± 0.03	0.37 ± 0.04	0.60 ± 0.05	0.67 ± 0.05	0.78 ± 0.06	0.65 ± 0.05

*p*		0.025	0.975	0.998	0.924	0.025	0.1	0.001^∗^	0.056	0.109

The table presents number of segments, average zHR, and standard errors for CC and non-CC segments, in each range of velocities. ^∗^Significant after correction for multiple comparisons [q(FDR) = 0.05].

#### Segment-by-Segment Analysis of Heart Rate and Subjective Togetherness (SRhigh)

We performed a similar segment-by-segment analysis to assess the difference in zHR between SRhigh and non-SRhigh segments with similar motion intensity.

In SRhigh segments compared to non-SRhigh segments, players move at higher velocities (SRhigh = 805.43 mm/s, non-SRhigh = 637.9 mm/s, non-parametric *p* < 10^-4^), and higher frequencies (SR = 1.18 Hz, non-SR = 1.14 Hz, non-parametric *p* < 0.01); and show higher z-normalized HRs (SR = 0.61, non-SR = 0.1, non-parametric *p* < 10^-4^).

**Figure 5**, **Tables 5** and **6** show the segment-by-segment analysis for nine Freq and nine maxVel bins. The same non-parametric procedure as in the previous section was applied to assess the statistical likelihood of the HR difference between SRhigh and non-SRhigh segments (see Supplementary Figures S9 and S10 for empirical control distributions). The results showed that in all Freq bins, and in eight of the nine maxVel bins, SRhigh segments had higher zHR than non-SRhigh segments, after correcting for multiple comparisons (q(FDR) = 0.05).

**Table 5:** zHR in bins of frequencies (Freq) in SRhigh and non-SRhigh segments.

Range of Freq (Hz)		0.25–0.5	0.5–0.75	0.75–1	1–1.25	1.25–1.5	1.5–1.75	1.75–2	2–2.25	2.25–2.5
SRhigh	#seg	340	346	257	163	235	138	158	92	230
	zHR ± SE	0.12 ± 0.04	0.45 ± 0.04	0.43 ± 0.06	0.2 ± 0.07	0.59 ± 0.06	0.9 ± 0.1	1.1 ± 0.09	1.27 ± 0.12	1.43 ± 0.07
Non-SRhigh	#seg	1618	721	613	543	645	400	455	309	778
	zHR ± SE	-0.1 ± 0.02	0.05 ± 0.04	0.11 ± 0.04	-0.05 ± 0.04	0.1 ± 0.04	0.19 ± 0.05	0.35 ± 0.05	0.38 ± 0.06	0.42 ± 0.04

*p*		<10^-4∗^	<10^-4∗^	<10^-4∗^	0.002^∗^	<10^-4∗^	<10^-4∗^	<10^-4∗^	<10^-4∗^	<10^-4∗^

^∗^Significant after correction for multiple comparison [q(FDR) = 0.05].

**Table 6:** zHR in bins of velocities (maxVel) in SRhigh and non-SRhigh segments.

Range of maxVel (mm/s)		0–250	250–500	500–750	750–1000	1000–1250	1250–1500	1500–1750	1750–2000	2000–2250
SRhigh	#seg	355	369	335	234	201	164	173	91	37
	zHR ± SE	0.47 ± 0.06	0.42 ± 0.05	0.33 ± 0.04	0.59 ± 0.07	0.72 ± 0.09	0.95 ± 0.09	1.02 ± 0.08	0.97 ± 0.09	0.81 ± 0.14
Non-SRhigh	#seg	1781	1530	896	529	441	323	222	167	193
	zHR ± SE	0.01 ± 0.02	0 ± 0.02	-0.02 ± 0.03	0.07 ± 0.05	0.16 ± 0.05	0.4 ± 0.06	0.65 ± 0.07	0.64 ± 0.07	0.68 ± 0.06

*p*		<10^-4∗^	<10^-4∗^	<10^-4∗^	<10^-4∗^	<10^-4∗^	<10^-4∗^	<0.001^∗^	0.002^∗^	0.192

^∗^Significant after correction for multiple comparison [q(FDR) = 0.05].

#### Combined Analysis and Summary

To further understand the effect of togetherness on zHR, while controlling for motion intensity, we performed an additional analysis that combined the Freq and maxVel dimensions, creating a grid of 9 (Freq) × 9 (maxVel) cells of motion intensity. In each cell, we computed the difference between the average zHR in togetherness and non-togetherness segments, and averaged this difference across cells. This analysis was done both for kinematically (CC) and for subjectively (SRhigh) defined togetherness. The statistical likelihood of the estimated average zHR difference was assessed using a non-parametric method. A bootstrapping procedure created *N* = 4,000 samples of shuﬄed data in each cell (ignoring the original classification), matching for original sample sizes in each cell. From this shuﬄed data, a distribution of control average zHR differences was computed. The empirical likelihood of the observed zHR difference between togetherness and non-togetherness segments was estimated directly from this distribution in the motion intensity plane. Only cells that contained a minimal number of segments (Ncell), out of 81 possible cells, were included. We tested the results for robustness by conducting this analysis for different Ncell values (20–60 segments, see Supplementary Figure S11).

The results showed that the observed zHR increases for subjectively defined togetherness (SRhigh) was highly significant (*p* < 0.001), regardless of the Ncell threshold. The results for the kinematically defined togetherness were less clear, exhibiting significant *p* values (*p* < 0.05) for Ncell < 32, and trend level likelihood (0.05 < *p* < 0.1) for Ncell > 32.

To summarize, the segment-by-segment analysis suggests that players’ cardiovascular activity is driven by two distinct sources. First, as can be seen in **Figures 4** and **5**, players’ HRs become faster as the velocities and the frequencies of their motion strokes increase. On top of that, motion segments marked by fine-grained kinematic synchrony (CC) or rated by both players as high in togetherness (SRhigh) exhibit additional HR elevations. The effect of subjective togetherness on players’ HRs was robust and invariant to motion characteristics, both when controlling for velocities and frequencies (**Tables 5** and **6**) and when combing these two motion dimensions. For kinematic togetherness, the effect was less clear, showing significant effects for the majority of frequency bins (**Table 3**) but only marginal effects when controlling for motion velocities (**Table 4**), and for combined frequency and velocity analysis.

### Kinematic and Subjective Indices Capture Different Aspects of Togetherness

To test the association between the two markers of togetherness we computed the 2 X 2 probabilities for each segment to be marked as CC or non-CC and as SRhigh or non-SRhigh. A goodness-of-fit Chi-square test showed a significant difference between the observed and the by-chance expected rate of segment marks [χ^2^(*N* = 8041) = 209.4, *p* < 10^-5^], with a small effect size (φ = 0.16, see Supplementary Table S1). Thus, an SRhigh segment is somewhat more likely to also be a CC segment.

The mean rate of SRhigh (0.20 ± 0.18) was substantially higher than the mean rate of CC (0.07 ± 0.08, see **Table 1**). To further explore the differences between these two measures we assessed the difference between CC rates and SRhigh rates for different velocity bins, using McNemar’s test for marginal frequencies (**Table 7**). As can be seen in the table, in the lower range of velocities, the proportion of SRhigh segments was higher than the proportion of CC segments, with the three lowest velocity bins showing a clear significant difference A reverse trend is seen for higher velocities (>750 mm/s), with a significant effect for the two medium velocity bins. A possible source for the difference between the two measures in low velocities are game periods in which players produce very little motion while still having an experience of togetherness. This analysis suggests that the two reported measures capture different aspects of togetherness.

**Table 7:** Proportion of CC and SRhigh segments in bins of maxVel.

Range of maxVel (mm/s)	0–250	250–500	500–750	750–1000	1000–1250	1250–1500	1500–1750	2000–2250	2250–2500
*#segments*	2136	1899	1231	763	642	487	395	258	97
CC	0.02	0.05	0.18	0.34	0.37	0.47	0.49	0.38	0.28
SRhigh	0.17	0.19	0.27	0.31	0.31	0.34	0.44	0.35	0.22
χ^**2**^	**240^∗∗^**	**190^∗∗^**	**34^∗∗^**	2.4	**5.35^∗^**	**18.5^∗∗^**	2.26	0.34	0.89

*#segments*: number of segments in this velocity bin; CC: proportion of segments with CC tag; SRhigh: proportion of segments with SRhigh tag. For each velocity bin, the higher of the two proportions is underlined. *χ^2^*, chi-square score from McNemar’s test. Significant differences are marked in bold. ^∗^*p* < 0.05, ^∗∗^*p* < 10^-4^.

## Discussion

In this study we investigated the physiological underpinnings of togetherness during joint motion improvisation. We measured temporally anchored kinematic, physiological and subjective responses of players, while they improvised motion together. We identified periods of togetherness using kinematic and subjective markers and assessed its individual and dyadic physiological characteristics. We next summarize and discuss the main results of the study.

### Periods of Togetherness in Joint Improvisation are Characterized by Increased Players’ Heart Rates

We used a previously developed kinematic marker of togetherness, which captures periods of fine-grained synchrony between the two players (CC motion). In game rounds with higher CC rates players exhibited increased HRs and produced motion at higher velocities and frequencies. A similar pattern was observed for a subjective marker of togetherness (SRhigh), which is based on players’ post-game dynamic ratings of experienced togetherness.

To show that the observed cardiovascular increases in game rounds marked by higher togetherness are not a by-product of the more intense motion during these rounds, we assessed players’ HRs in motion segments, while controlling for motion intensity.

For the subjective marker of togetherness, a robust effect was observed (**Figure 5**): players exhibited increased HRs in togetherness periods regardless of motion intensity. For the kinematic marker of togetherness, a marginal trend was observed for this effect (**Figure 4**). Taken together, these results suggest that periods of togetherness during joint motion improvisation, and in particular periods which are subjectively experienced as togetherness, are accompanied by increased cardiovascular activity.

The observed cardiovascular increases during moments of togetherness can underlie the engaging and rewarding experiences reported by performers in peak moments of joint improvisation (Berliner, 1994, p. 389). Interestingly, similar findings arise in recent studies of the physiology of flow. Flow is a unique state of optimal performance, full involvement and enjoyment (Peifer, 2012). Sawyer suggested that peak moments in joint improvisation can be considered as examples of group flow (Sawyer, 2003, 2008). Flow and togetherness are similar – both involve optimal performance, are highly engaging and rewarding (Berliner, 1994, p. 389) and evoke a subjective description of blurring of the boundaries between the self and the task (in flow) or the self and others (in togetherness). A number of studies, investigating the physiological processes during flow, repeatedly demonstrated that flow states are associated with increased sympathetic activation (Kivikangas, 2006; de Manzano et al., 2010; Mansfield et al., 2012; Gaggioli et al., 2013; Peifer et al., 2014; Ulrich et al., 2014). These findings were suggested as evidence for a state of effortless attention arising through the interaction of positive affect and high attention (Bruya, 2010; de Manzano et al., 2010; Peifer, 2012). The similarity between periods of togetherness in joint improvisation and group flow, suggests that the increased cardiovascular activity found in our study manifest a psychological state of enhanced engagement and enjoyment. Further research is needed to better understand the physiological mechanisms driving the increased cardiovascular activity observed in this study (e.g., whether it indicates states of sympathetic activation) as well as the psychological states associated with it.

### Periods of Togetherness in Joint Improvisation might be Characterized by Increased Inter-Player Correlation of the Cardiovascular Activity

An additional physiological marker that was investigated in this study is the correlation of players’ HRs. Temporal alignment of physiological activity across communicating individual, has recently acquired substantial research attention and support (Hasson et al., 2012; Konvalinka and Roepstorff, 2012). In particular, a series of studies suggested that increased physiological synchronization across individuals marks distinctive states of interpersonal coordination and connection (Butler, 2011; Helm et al., 2012; Konvalinka and Roepstorff, 2012; Hari et al., 2013).

We found positive association between correlation of players’ HRs and the rate of togetherness, defined both kinematicaly and subjectively (**Table 2**). However, the interpretation of this finding should be done with caution, as we also observed positive correlations between inter-player HR correlation and the intensity of their motion. Accordingly, the observed association between togetherness and physiological alignment could be a side-effect of lower level, motion-physiology linkage. The effect of motions on physiological responses (such as increased HR as a result of performing faster movements, **Figures 4** and **5**) poses a challenge for physiological research of joint improvised motion. Future studies are needed to disentangle the low-level (within-person) motion-physiology linkage and the across-person synchronization of physiological activity, possibly associated with distinctive interpersonal states, such as togetherness.

In addition, it might be interesting to investigate physiological coupling during joint improvisation employing other measures, such as respiration. As an anecdote we mention a practice from Playback Theater training: before performing a long mirroring sequence by three or four actors (Lubrani, 2009), the actors are instructed to ‘breathe together for 10–20 s.’ Breathing in-sync seems to help producing more coherent, and more complex, mirror-like motion. It will be interesting to see if induced physiological synchronization can lead to enhanced behavioral synchrony.

### A Subjective Post-Game Measure Enriches the Description of Togetherness Moments

In this study we introduced a new measure of togetherness based on post-performance SR by the players. *Being in the zone* was described by experienced improvisers as “a state of unselfconscious awareness” (Seham, 2001). We therefore assumed that it would be difficult for players to report on their subjective experience during the game without disrupting it, and used a dynamic post-game subjective report as the next best thing. We compared the kinematic (CC) and the subjective (SRhigh) markers of togetherness and showed some agreement between them – CC segment is more likely to be in SRhigh segment (and vice versa) than expected by chance.

The CC and SRhigh measures are different: the CC measure is well-defined and is theory-based (the lack of reactive behavior of the two players, see Noy et al., 2011; Hart et al., 2014). The SRhigh measure is subjective and intuitive, and it is not clear what sources of information are used by the players to complete the togetherness rating. While previous studies report a clear association between kinematic synchrony and the experience of social connection (e.g., Hove and Risen, 2009; Valdesolo and DeSteno, 2011), the current study suggests that there is no one-to-one correspondence between these two measures. We suggested one possible source to the difference between these two measures: the CC measure was under-performing in low velocities (see **Table 7**). In the extreme low velocity, several players reported having moments of togetherness in game periods without any motion (see example in **Figure 2**).

The CC detector, which is based on the kinematics of the motion tracks, might therefore capture only a slice of the rich interaction happening in the mirror game. Future research can better understand the differences between these two measures of togetherness, for example by a detailed analysis of the game periods that were marked differently by the two measures of togetherness reported here.

Another interesting avenue for future research is studying what visual features drive the SR of togetherness. For example, are these ratings driven by particular non-verbal cues, such as players’ gazes and facial expressions? To the extent that togetherness periods are associated with positive affect and high engagement, as suggested above, the SR might be based on identification of such moments during the game.

The SRhigh measure is a dyadic measure, describing game periods where both players provided high rates of togetherness. Over all, there was moderate agreement between the two players. However, in some cases there was high disagreement between players’ judgments. This finding can be discussed in light of recent studies on the level of post-performance agreement between performers (Wöllner, 2013; Schober and Spiro, 2014). Studying improvising jazz musicians, Schober and Spiro (2014) employed a novel post-performance interviewing technique, and showed a moderate to low agreement between the two improvisers, with instances of high disagreements on what happened in a particular moment of the performance. Future studies can better understand the level of agreement between improvising dyads using different methods, as well as comparing self-reports of the performers to reports from external viewers, and to quantitative measures, such as the CC motion used in this study.

## Conclusion

Taken together our results demonstrate that during joint improvisation players enter a unique dyadic state, in which they produce highly synchronized motion, feel together with the other player and exhibit increased cardiovascular arousal. We also provide initial evidence that this state might be accompanied by increased alignment of players’ HRs.

The current study shows the feasibility of studying an open-ended creative coordination task, by incorporating on-going physiological, kinematic and subjective measures. This approach has a potential to contribute to the science of performance in general, by studying the fully embodied performance process that unfolds in time. An especially intriguing possibility is to study on-stage performance, while incorporating real-time and post-performance measures. This can be extended beyond the stage to include measurements of audience members. Combing temporal measures of performers and spectators can provide insights on the optimal conditions for the emergence of togetherness, and the dynamics of entering (and leaving) the ‘zone.’

## Conflict of Interest Statement

The authors declare that the research was conducted in the absence of any commercial or financial relationships that could be construed as a potential conflict of interest.
