# Supplementary material for: Being in the zone: physiological markers of togetherness in joint improvisation
Source: Front Hum Neurosci. 2015 May 5;9:187. doi: 10.3389/fnhum.2015.00187 (PMC4419713; doi:10.3389/fnhum.2015.00187)
Supplement: Supplementary file 1 [file Data_Sheet_1.DOCX]

***Supplementary Material***

**Being in the zone: physiological markers of togetherness in joint improvisation**

**Lior Noy^1,2^*, Nava Levit-Binun^3^, Yulia Golland^3^**

^1^Department of Molecular Cell Biology, Weizmann Institute of Science, Rehovot, Israel
^2^The Theatre Lab, Weizmann Institute of Science, Rehovot, Israel
^3^Sagol Institute for Brain and Mind , Baruch Ivcher school of Psychology, Interdisciplinary Center (IDC), Herzliya, Israel

***Correspondence:** Lior Noy, Department of Molecular Cell Biology, Weizmann Institute of Science, Rehovot, Israel

[lior.noy@weizmann.ac.il](mailto:lior.noy@weizmann.ac.il)

1. **Supplementary Figures and Tables**

## Supplementary Tables

**Supplementary Table 1. Comparing the joint distribution of CC and SRhigh segments.** Each motion segment was marked as CC or nonCC (kinematic togetherness), and as SRhigh or nonSRhigh (post-game dyadic report of subjective togetherness). The table presents the observed ratio (right) computed from the combination of these two tags for each segments, and the expected ratio, computed from the marginal probabilities (taking into account only one tag at a time). A goodness-of-fit Chi-square test showed a significant difference between the observed and the by-chance expected rate of segment marks (χ^2^(N=8041) = 209.4, *p* < 10^-5^ ), that is, a CC segment is more likely to also be an SRhigh segment (and vice versa) than expected by the marginal distributions. However, notice that the ratios are quite similar, and that the effect-size, φ = 0.16 is considered small.

| **Expected**  **Ratio** |  |  |  |  | **Observed Ratio** |  |  |  |
| --- | --- | --- | --- | --- | --- | --- | --- | --- |
|  | notSRhigh | SRhigh |  |  |  | notSRhigh | SRhigh |  |
| nonCC | **0.62** | **0.20** | 0.82 |  | nonCC | **0.65** | **0.17** |  |
| CC | **0.13** | **0.04** | 0.18 |  | CC | **0.11** | **0.07** |  |
|  | 0.76 | 0.24 |  |  |  |  |  |  |

## Supplementary Figures


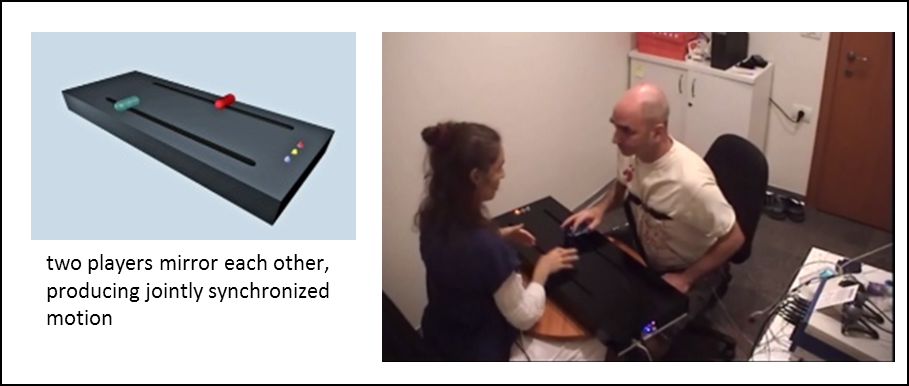


**Supplementary Figure S1: Integrating the one-dimensional mirror game setup with physiological measurements.** The mirror game setup (left panel, schematic drawing) was developed in previous studies (Hart et al., 2014; Noy et al., 2011) to study joint improvised motion. The mirror game was integrated with dyadic physiological measurement setup (right panel), which allows to measure simultaneously physiological activity of two people. Recording of motion tracks (50 Hz) from two players was synchronized with recording (1 kHz) of their cardiovascular activity (ECG) via dedicated TTL trigger.

**
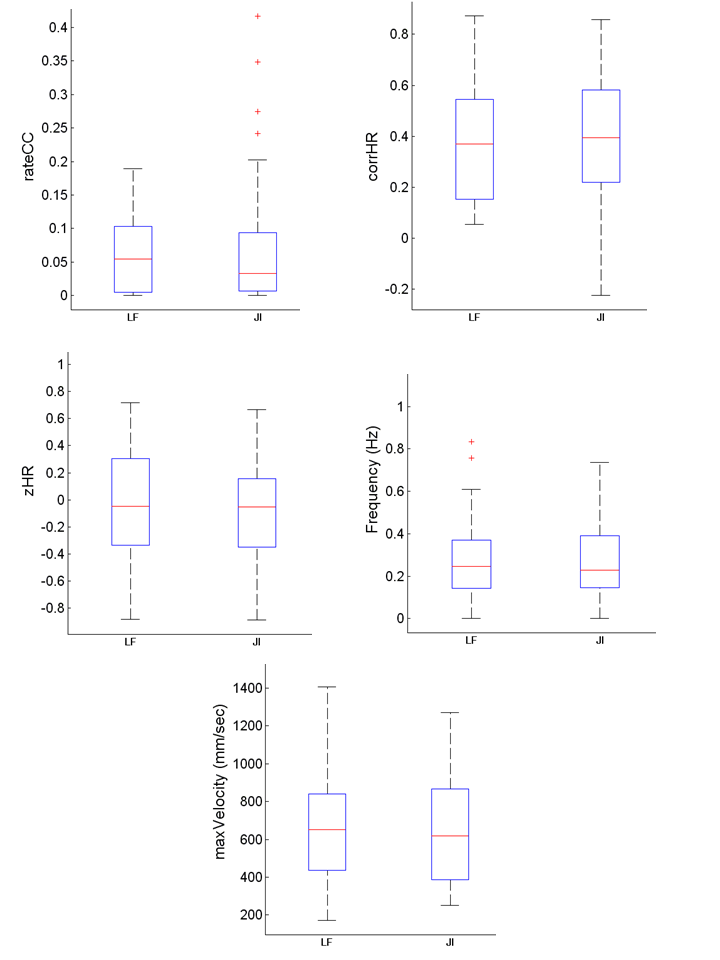
**

**Supplementary Figure S2. Kinematic and physiological measures are similar in Leader-Follower and Joint-Improvisation rounds.** We compared the game characteristics (see Table 1 in the main text) in the two types of rounds: Leader-Follower (LF), when one player is designated as the leader of the common motion, and Joint-Improvisation (JI), when there is no designated leader or follower. In the current experiment only the first two rounds (out of six) where LF rounds. Shown are box-whisker plots for the five measures described in the main text. For each measure, we pooled the values over all LF rounds (N=24) and all JI rounds (N=53) in our dataset (data from some rounds were missing due to measurement errors, see Methods). Red line - median, box - 25th to 75th percentiles, whiskers - the most extreme data points not considered as outliers, red crosses – outliers. CCrate – rate of CC motion in each round. corrHR - within dyad heart rate correlation. zHR - median of z-normalized heart rate. maxVelocity – weighted median of maximal velocity. Frequency - weighted median of frequency. For zHR, maxVelocity and Frequency round data is averaged between the two players. In all five measures there were no statistical differences between LF and JI rounds (two-samples *t*-test, CCrate: *t*(75)=-0.35, N.S; corrHR: *t*(75)=-0.26, N.S; zHR: *t*(75)=0.43; N.S.; maxVelocity: *t*(75)=0.48, N.S.; Frequency: *t*(75)=0.84, N.S.).


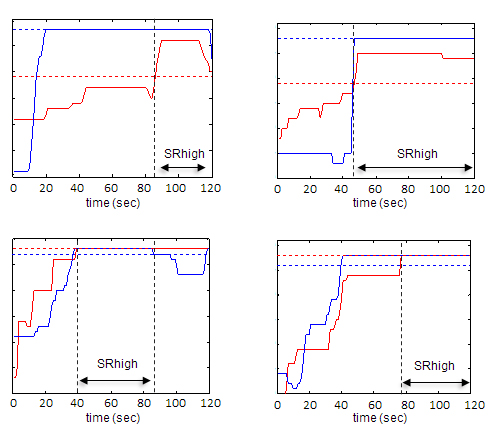


**Supplementary Figure S3. Dyadic measure of subjective togetherness.**

For each game round we identified periods in which both players provided high rates of togetherness (SRhigh). To account for individual differences in the range of subjective responses (SR), we assessed the SRhigh threshold for each player individually (top 30% of all sample points). We calculated a dyadic measure of high subjective togetherness (SRhigh), by indexing all data periods in which both players’ SRs were above threshold. Here we present examples of individual SRs (player 1 – blue line, player 2 – red line, arbitrary units) in different pairs and different game rounds. These examples demonstrate that 1) players had different response ranges, therefore different thresholds (marked by red/blue dotted lines) 2) players served as ‘control’ to each other, suggesting that dyadic measure of SRhigh is a more reliable index then individual measures.


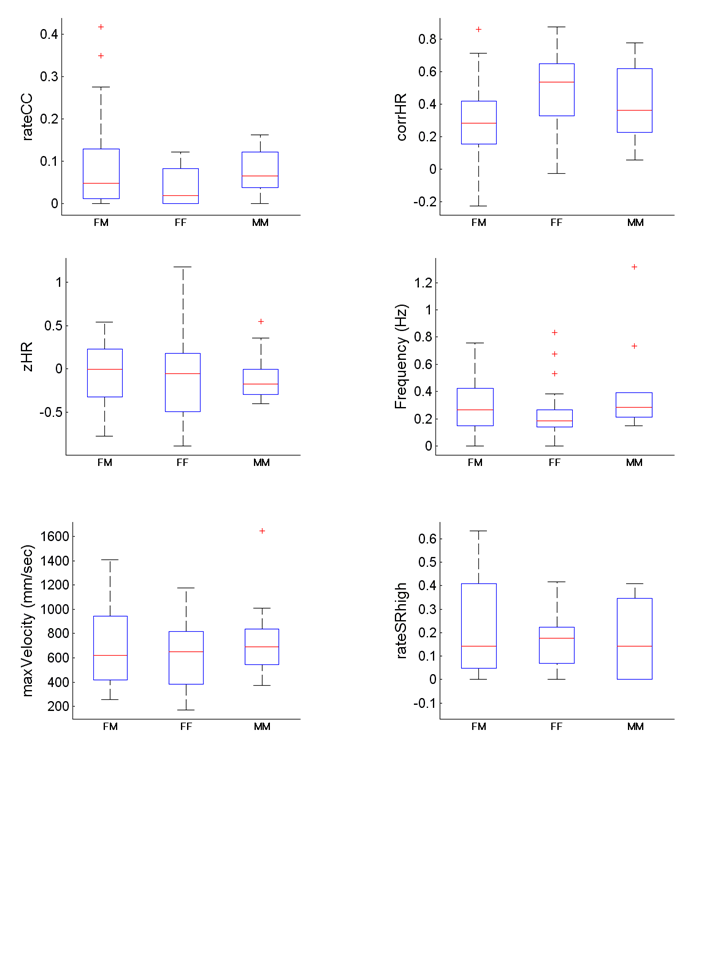


**Supplementary Figure S4. Kinematic and physiological measures show some differences between different gender pairings.** We compared the six kinematic, physiological and subjective measures, between rounds in different gender pairing: female-male (FM, 3 pairs); female-female (FF, 5 pairs); male-male (MM, one pair). Shown are box-whisker plot, computed over all rounds of a particular gender pairing (see previous figure for five first legends, and rateSRhigh – ratio of round were both subjects had high Subjective Rating). To statistically explore possible gender-pairing effects on the physiological and kinematic characteristics, we conducted a Mixed Repeated Measures Linear model analysis on the five measures using gender (with FM and FF as a fixed effect factor, excluding the single MM pair). Only rateCC and corrHR showed significant gender-pairing effects (p<0.05 and p<0.001 for rateCC and corrHR respectively), with FM pairs showing somewhat higher rateCC and lower corrHR than FF pairs. Due to the small number of pairs involved in this analysis, we report this result here and do not discuss it further in the main text.

**
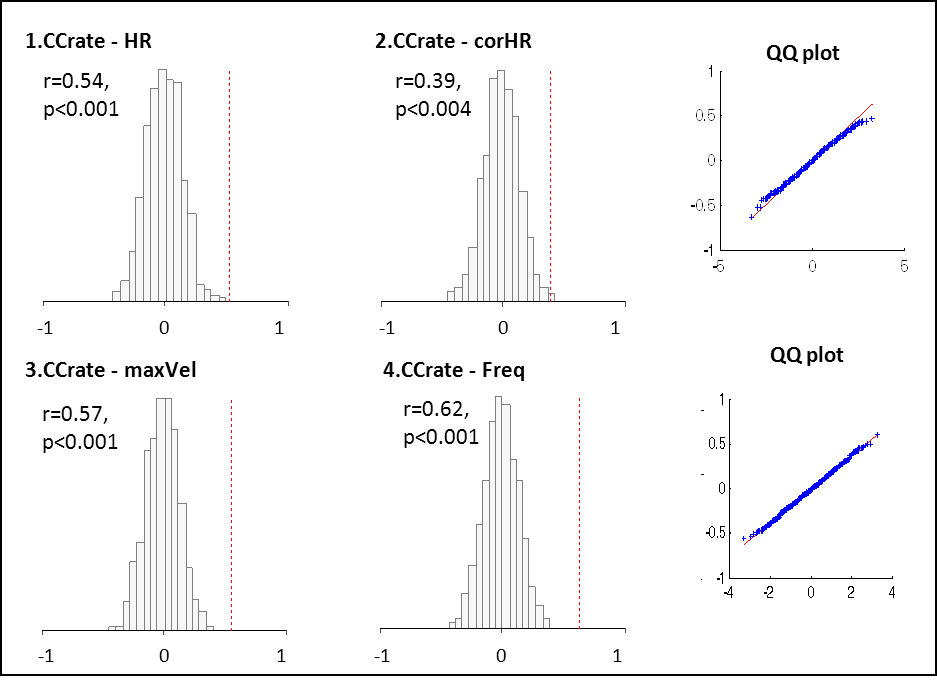
**

**Supplementary Figure S5. Control distributions for assessing the likelihood of observed correlations between kinematic togetherness (CCrate), physiology and motion intensity.** To assess the statistical likelihood of the observed correlations between kinematic togetherness (CCrate), physiological characteristics (zHR, corrHR), and motion intensity (maxVel, Freq), we applied a non-parametric bootstrapping procedure. For each pair we created an empirical control distributions of correlation coefficients (*r*s) by shuffling the order of rounds (n=1,000, with repetitions). These control *r*s were averaged across pairs, providing sampling control distributions. The group-wise experimental rank correlations (marked by dotted red line) fall in the right tail of the control distributions. p values were estimated directly from the control distributions.


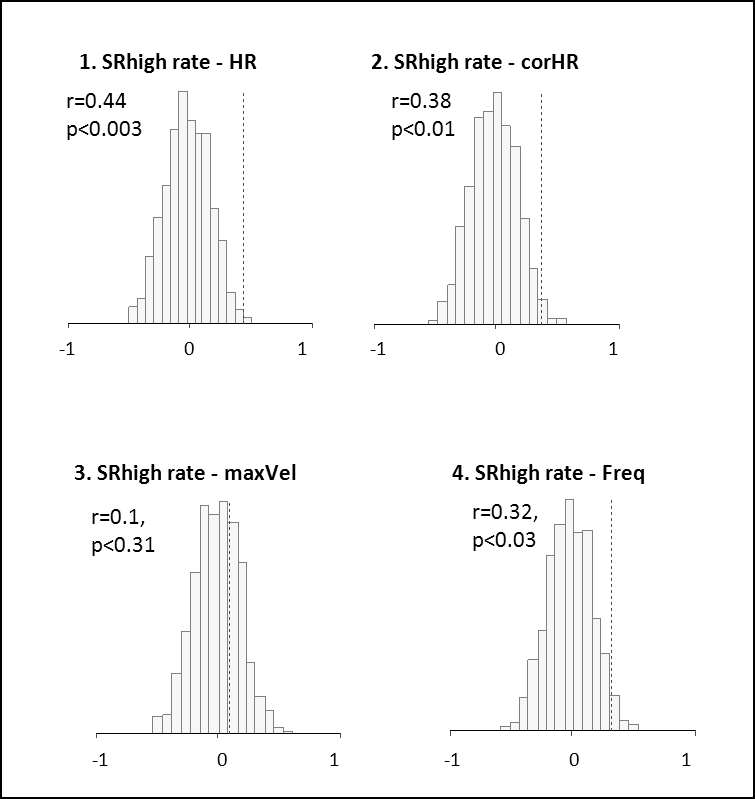


**Supplementary Figure S6. Control distributions for assessing the likelihood of observed correlations between subjective togetherness (SRhigh rate), physiology and motion intensity.** To assess the statistical likelihood of the observed correlations between ‘subjective togetherness’ (SRhigh) and 1, 2 - physiological characteristics (HR, corrHR) and 3,4 -motion intensity (maxVel, Freq), we applied non-parametric bootstrapping procedure. For each pair we created empirical control distributions of the correlation coefficient (*r*s) by shuffling the order of rounds (n=1,000, with repetitions). These control rs were averaged across pairs, providing sampling control distributions. *p values* were estimated directly from the control distributions.


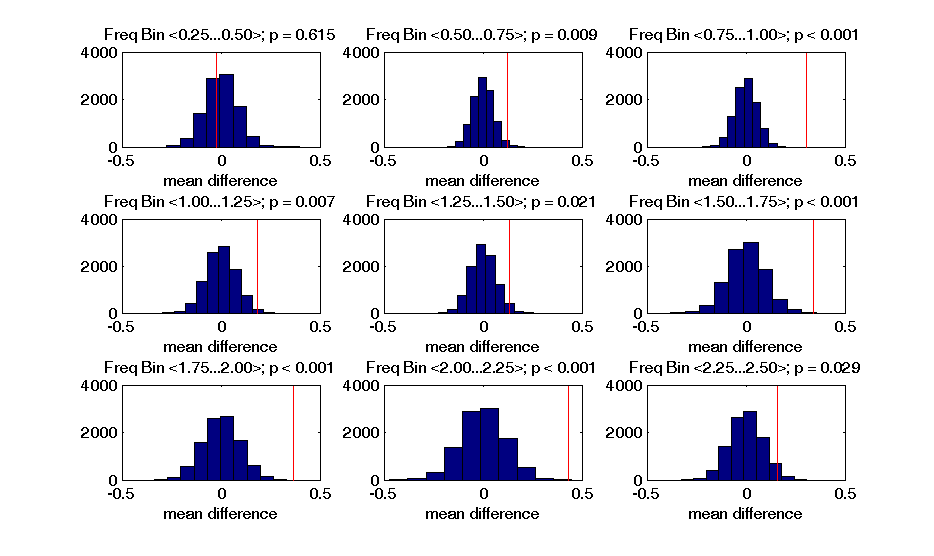


**Supplementary Figure S7. Statistical comparison of zHR in CC and non-CC segments, in the same Frequency bins.**  Each subplot show the comparison of the real mean-difference of the zHR of CC and non-CC segments in one frequency bin ( mean(zHR(CC)) – mean(zHR(non-CC)) ) to an empirical distribution of mean-differences produced using bootstrapping. To create this distribution we sampled (with replacement) 10,000 simulations of the “CC” and “nonCC” group of segments, in the same size as the original groups. These segments were sampled regardless of the CC/non-CC tags, that is, using shuffled data. Red line – actual mean-difference. Blue distribution – a histogram of the simulated mean-differences. The resulting *p* value (in the title of each subplot) is the ratio of this distribution that is larger than the actual mean-difference.


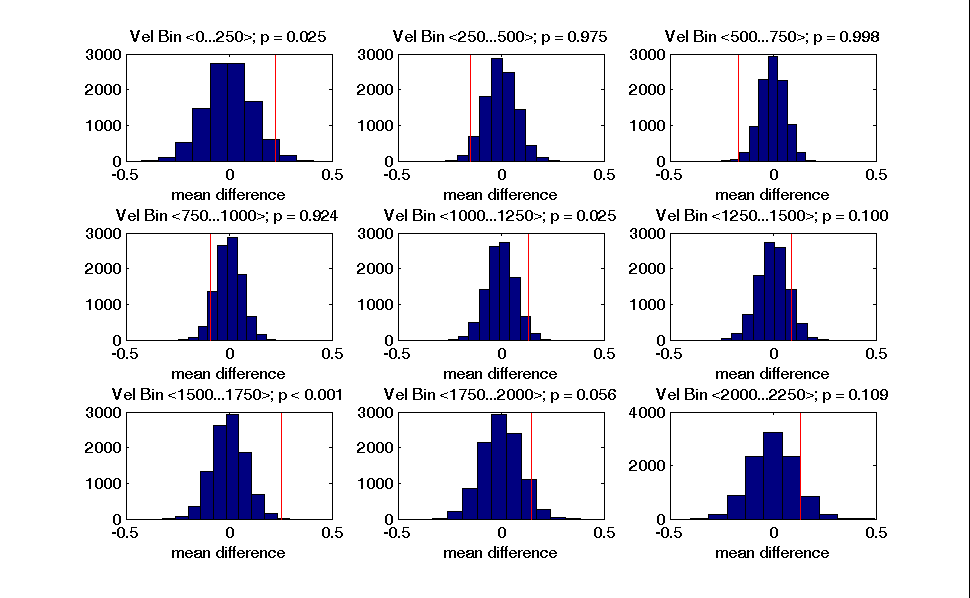


**Supplementary Figure S8. Statistical comparison of zHR in CC and non-CC segments, in same Velocity bins.**  Similar to Figure S7.

­­

**
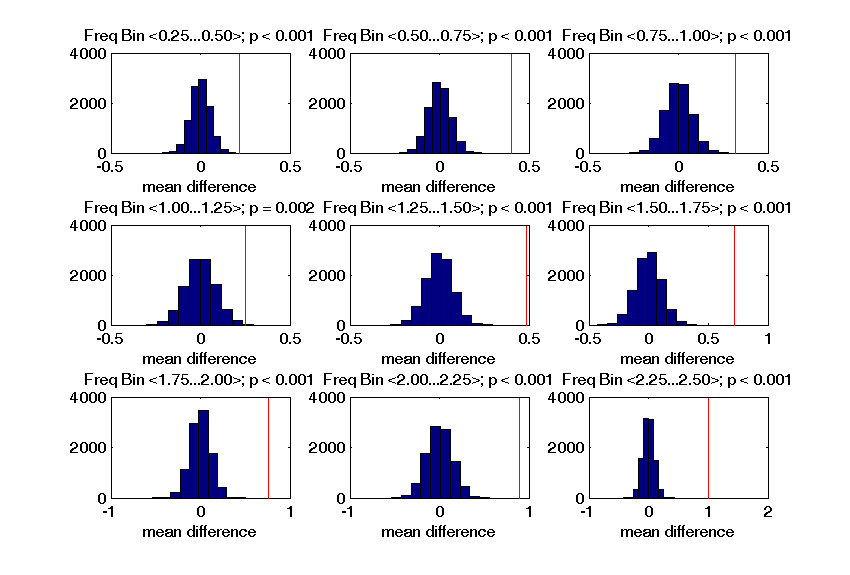
**

**Supplementary Figure S9. Statistical comparison of zHR in SRhigh and non-SRhigh segments, in same Freq bins.**  Similar to Figure S7.

**
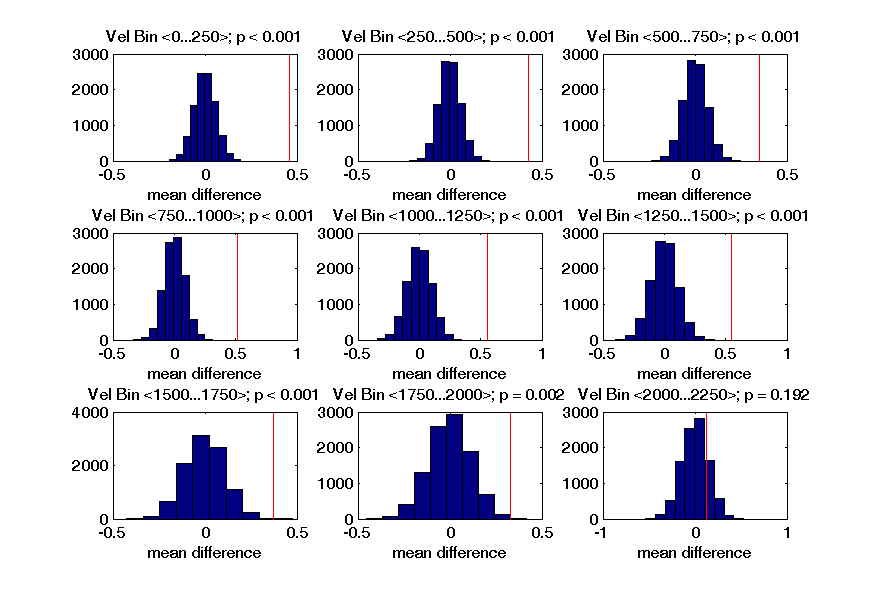
**

**Supplementary Figure S10. Statistical comparison of zHR in SRhigh and non-SRhigh segments, in same maxVel bins.**  Similar to Figure S8.

**
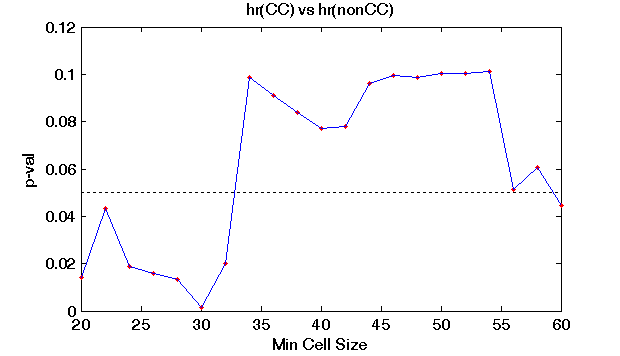
**

**Supplementary Figure S11. Analyzing the main result on the maxVel/Frequency grid, with a different minimal number of segments per cell.** The figure shows a sequence of comparisons of comparing HR in CC vs. nonCC segments over a grid of 9*9 cells of maxVel/Frequency. In each comparison, only cells that have at least Min-Cell-Size segments for both classes (CC and nonCC) were included in the analysis. Min-Cell-Size was in the range of [20..60], resulting in [37..11] included cell (out of 81) for the CC analysis.

For each value of the parameter, a bootstrapping procedure created N=4,000 samples of shuffled data in each cell (ignoring the original CC/nonCC labels), at the same size as the original two groups in that cell. From this shuffled data, a distribution of simulated difference between the groups (the difference of average of classes in each cell, averaged over all relevant cells) was computed, and compared to the real difference, to create the empirical *p*-value (the number of simulated samples above the real difference). For the CC classification the empirical *p*-value fluctuates around 0.05, and the result is not clearly significant with the current sample size.

In contrast, for the SRhigh classification the difference was robust over range of Min-Cell-Size values. The same procedure as above was repeated. For Min-Cell-Size at the range of [20..60], there were [33..4] cell for the SRhigh analysis (there are fewer segments as the subjective post-game analysis was done only on six rounds for each pair). Regardless of the minimal number of segments per cells, the bootstrapping procedure resulted with p < 10^-4^.

1. **References**

Hart, Y., Noy, L., Feniger-Schaal, R., Mayo, A. E., and Alon, U. (2014). Individuality and Togetherness in Joint Improvised Motion. *PLoS ONE* 9, e87213. doi:10.1371/journal.pone.0087213.

Noy, L., Dekel, E., and Alon, U. (2011). The mirror game as a paradigm for studying the dynamics of two people improvising motion together. *Proc. Natl. Acad. Sci.* 108, 20947–20952.
